# The “ELDERDES” model: how older adults' adoption of digital environment solutions improves residential experiences, health outcomes, and enables independent living

**DOI:** 10.3389/fpubh.2026.1724293

**Published:** 2026-02-03

**Authors:** Stephen M. Golant

**Affiliations:** Department of Geography, University of Florida, Gainesville, FL, United States

**Keywords:** aging in the right place, digital destinations, digital environment, digital literacy, health stressors, healthspan-lifespan gap, gerontechnology, optimal aging

## Abstract

Global increases in longevity have led to a rapidly growing population of older adults entering advanced old age, facing increased risks of chronic illness, mobility limitations, sensory impairments, cognitive decline, poor emotional health, and social losses. These vulnerabilities make it more difficult for them to maintain independent living arrangements and to age optimally in their current homes, neighborhoods, and communities. Routine tasks like climbing stairs, reaching high shelves, or avoiding slippery floors become unsafe. Older people struggle to reach inhospitable community destinations to meet their obligatory and discretionary needs. As their “activity spaces” shrink to the home's confines, they experience new health stressors that further complicate age-related declines. Research and intervention strategies have emphasized making homes age-ready and reducing barriers to community access. However, rapid advances in digital technologies necessitate a reevaluation of how older adults interact with their surroundings. Homes can function as digital environments, equipped with internet-connected devices and AI-powered software. Interior sensors can detect dangerous temperatures, and wearables can monitor vital signs and medications. Motion detectors can summon emergency responses in the event of falls or other medical emergencies. Older adults can access virtual or “digital destinations” in real time—to buy goods, order food, use patient portals, access financial or government services, and engage in social or recreational activities. We are witnessing a paradigm shift in how older adults can maintain their independence and age in place. Their homes have transformed into environmental monitoring hubs and digital control centers, increasing their safety and health and reducing their dependence on neighborhood and community resources. Although older people are increasingly adopting these solutions, many remain reluctant. This provides the rationale for the ELDERDES model, which identifies the factors that influence older adults' adoption of digital environment technologies and how four categories of digital environment solutions (DES) contribute to their positive residential experiences, improved health outcomes, independent living, and optimal aging. There are many reasons to argue that increasing older people's access to their digital environments will become a more salient arena for research and intervention than primarily adapting the features of their traditional physical or social environments.

## Introduction

Global increases in life expectancy have led to a steadily growing population of older adults in advanced ages—often referred to as “Fourth Age” elders (1). Although older adults are living longer chronologically (lifespan), they spend fewer years in good health (healthspan). This “healthspan-lifespan gap,” averaging just under 10 years worldwide, is larger for women than for men and is widening in some regions (2). During this vulnerable time, older adults face higher risks of chronic illnesses, mobility limitations, sensory impairments, and cognitive decline (3). Their social networks also shrink as spouses, family members, and longtime friends die, fall ill, or move away, and as late-life divorces occur (4).

As they seek effective ways to cope with these declines and losses, they begin to reevaluate whether their current homes, neighborhoods, and communities remain comfortable, enjoyable, and safe places that are conducive to independent living. Such concerns are justified. Homes present new challenges. Negotiating stairs, high shelves, and slippery bathroom floors becomes harder and less safe. Especially at risk are older people who live alone or with another vulnerable partner, who worry about getting help after a serious fall or medical crisis. Traveling to outside destinations to meet daily needs also becomes more difficult. Traditional transportation modes—such as cars, transit, or walking—may require excessive energy to use, feel unsafe, or be unavailable (5, 6). As their “activity spaces” shrink to the confines of the home, older adults struggle to shop, manage finances, access healthcare, supportive services, worship, socialize, and pursue recreational activities (7). Their increasingly incongruent residential environments create new health stressors that further complicate those age-related health declines (8). Overall, empirical research emphasizes that these individual losses, incongruent residential experiences, and new health stressors take a toll on their emotional health and act as barriers to aging well (9).

Taking a more optimistic view, this paper argues that older adults have new and increasingly effective ways to cope with these individual and environmental adversities. Advancements in digital technology and artificial intelligence (AI) now enable health- and mobility-challenged older people to make their homes safer and physically and emotionally more responsive to their needs. It is now feasible for them to access a wide range of essential and discretionary goods, services, information, and social relationships without leaving their dwellings.

Given the potential benefits of these digital solutions, understanding how older adults decide to adopt these options is a crucial area of study. Although acceptance rates are rising, an old-young digital divide persists, with older people lagging behind younger populations in both their acceptance of these approaches and their comfort using them.

This paper develops a conceptual model that addresses this need and has two main objectives. The first is to demonstrate that older adults can benefit from up to four categories of digital environment solutions (DES) that help them proactively address or manage their mismatched housing experiences, health-related issues, and threats to independence, and age optimally.

Second, it examines how personal differences among older adults and their varying subjective evaluations of these options both encourage and limit their acceptance. Striving to offer a comprehensive understanding of these deliberations, the model shows that when deliberating about adopting these digital solutions, they can pursue up to six decision-making pathways.

The proposed decision-making framework builds on early technology adoption models such as TAM, UTAUT, and Elderadopt along with related empirical research, but distinguishes itself from these earlier efforts in several important ways (10–15).

Most importantly, the conceptual model proposes that the digital environment older adults can introduce into their homes comprises two interconnected but distinct components: its digital equipment and its digital destinations. The digital equipment includes broadband connections, sensors, computers, smartphones, voice-activated personal assistants, and wearables integrated with software applications and online platforms powered by artificial intelligence (AI). This equipment records, stores, and analyzes information about a dwelling's interior environment and monitors older residents' activities and health conditions, relaying this information directly to users or remote healthcare providers. Emerging digital devices—including animal-like and humanoid robots—extend these capabilities by providing companionship, emotional support, and assistance with household tasks. Secondly, digital destinations—a category rarely treated separately—are the virtual or online places and spaces that older adults access through voice commands, taps, or motions to get information, buy goods, see doctors, check lab results, access financial and governmental services, get entertained, and connect socially.

These digital components vary in physical form and functionality; using them requires different mental and physical skills. It is essential to recognize these different components because older adults often evaluate their benefits and usability differently, with some being more efficacious and easier to use.

As with previous models, we propose that individual differences, such as socioeconomic status, cultural background, resilience, and health issues, influence older people's decisions about adoption. However, this model also distinguishes digital literacy as a new and salient indicator of personal competence, which, unlike many other individual attributes, can be modified with appropriate interventions.

The model also argues that understanding older people's decision to adopt these digital solutions requires more than focusing solely on these options. When they subjectively assess the effectiveness and usability of new technologies and consider their unintended downsides, older adults often compare them with the benefits and drawbacks of more traditional products, services, care, and travel alternatives.

The potentially broad and positive impacts of these digital technology solutions on the quality of life of older people highlight the need to realign research and policy priorities, which have mainly focused on the age-ready design features of dwellings and overcoming neighborhood and community barriers that restrict travel access. Encouragingly, research on the role of digital technologies in later life is expanding (16, 17). Environmental gerontologists, for example, have widened their focus, with the Context Dynamics in Aging (CODA) framework explicitly incorporating technological alongside physical and social contexts (18). Scholars have also called on the World Health Organization to broaden its age-friendly initiatives to include digital and communication technologies (19). Nevertheless, relatively few rigorous conceptual frameworks have fully articulated how these digital innovations affect older people's housing experiences and health outcomes (20).

## Literature's perspective on older people's residential environments

A substantial body of research across geography, sociology, architecture, planning, and environmental gerontology shows that many older adults—especially those in advanced age—feel their homes and apartments are poorly suited to their changing needs. It focuses on how occupying and using these living environments worsen physical and mental health outcomes by hindering personal functioning and growth, making independent living more difficult, and reducing social opportunities (18, 21). Although older people still strive to age in place, many experts and professionals argue that they should transition to more supportive housing and care options (8).

Traditionally, this literature has concentrated on the unaffordability and physical issues (e.g., water leaks, roof defects, structural damage) of older people's homes. Those with lower incomes and members of minority groups are at greater risk. Studies also focus on how older people's homes often lack age-appropriate features. This leads to more hazardous indoor environments (e.g., fall risks, extreme heat or cold), which increases the likelihood of accidents and health issues, such as hypothermia or hyperthermia (22).

Complementing this body of research is substantial evidence of the adverse outcomes experienced by older people interacting with their neighborhood and community settings. It highlights the travel difficulties that vulnerable older adults face when attempting to reach destinations outside their homes. Sensory and cognitive impairments can significantly impair driving abilities, especially at night or in heavy traffic (23). The problem has worsened because older adults now primarily live in low-density, automobile-dependent communities characterized by dispersed commercial areas and limited public transportation (24). Even physical proximity is no guarantee of access. Physical fatigue and unsteady gaits can hinder walking, particularly when older individuals are carrying packages, dealing with adverse weather conditions, negotiating heavy traffic, or navigating the large interiors of major retail box stores. These findings reaffirm long-standing planning concepts, particularly the “friction of space,” which describes how distance and environmental constraints significantly hinder older adults' ability to access essential goods, services, social connections, and information.

Older adults often perceive their external physical and social worlds as hostile and physically or socially inaccessible (23, 25). Mobility limitations make it challenging to navigate inhospitable land uses, such as poorly designed walkways, the absence of seating (e.g., benches), quickly changing traffic signals, and crime threats. Many older adults feel upset about displaying their vulnerabilities—for instance, using canes or walkers to manage slower, unsteady walking—because they fear the condescending judgments of others. They also encounter ageist assumptions and patronizing behaviors from sales and service staff (26). Consequently, for many older adults, traveling to community destinations often reinforces feelings of incompetence and powerlessness (25).

Older people are also facing a new challenge when accessing their community's resources (27). More organizations, businesses, events, and activities are now only accessible online. For example, accessing healthcare information, physician portals, financial services, government benefits, restaurant reservations, and even library books now increasingly requires some modicum of digital literacy. Consequently, older adults—especially the more vulnerable—confront an even greater risk of being unable to access an even larger share of their everyday needs, which is clearly detrimental to their physical and mental health. The old-young digital divide becomes even more relevant.

## New health stressors

Older people often confront new health stressors due to these incongruent residential experiences. Broadly interpreting their possible health outcomes (28, 29), six different types of new problems can arise:

- **Worsening physical health outcomes**. Limited access to essential destinations outside their homes creates new health risks. Examples include poor nutrition (30) (due to difficulties shopping for food), undiagnosed health conditions (from missed medical appointments) (31), greater risks of falling (from unsafe walking environments) (32), and increased feelings of pain, fatigue, or weakness (because of travel challenges) (23).
- **Worsening psychological health outcomes**. Living in incongruent housing circumstances makes older adults more aware of their physical limitations and their inability to do once-easy tasks. Negative psychological health symptoms often result from recognizing these vulnerabilities. For instance, knowing others see them as mobility-impaired damages their self-esteem; less interaction with friends can lead to depression and anxiety; lack of easy access to professional counseling can worsen mental health (33); fewer recreational and leisure activities can lead to lost cognitive stimulation benefits (34); negotiating busy intersections triggers anxiety; and, not having access to green or natural spaces removes a source of enjoyment and stress-relief (35). Constantly facing mobility barriers also causes older people to feel less in control of their lives and activities. Examples include losing the ability to drive or climb stairs in one's own home.
- **Worsening social health outcomes**. Being confined to their homes due to mobility limitations makes it increasingly difficult to visit neighbors, friends, or nearby family. This social isolation increases the likelihood of experiencing loneliness and feeling emotionally unsupported. Feeling lonely, in turn, is linked to a higher risk of experiencing an accident alone at home, decreased motivation to cook, unnoticed health changes, increased vulnerability to financial scams, reduced mental stimulation, and depressed feelings about one's life circumstances (36).
- **Unhealthy dwelling-centered lifestyles**. Restricted activity spaces cause older people to spend more time indoors and, in severe cases, to become homebound (7). Confinement to the home with more limited external information, stimulation, and emotional support increases the risks of depression, anxiety, and, more generally, reduced optimism about aging well (37, 38).
- **Greater reliance on external help**. When older adults are unable to perform their usual activities, they tend to rely more on family or paid caregivers. While this addresses immediate concerns about maintaining independence, it may also reduce their long-term feelings of autonomy, competence, and self-worth, and increase depressive symptoms (39, 40).
- **Challenges to preferred aging-in-place**. Older persons fear that their negative residential experiences will eventually force them to abandon their goal of living independently. They fear the prospect of moving into relatives' homes or entering long-term care. Such moves would sever their connection to familiar homes, routines, and social networks, causing them to lose their strong sense of place and its emotional benefits (41).

## Supportive theoretical perspectives

Theoretical literature from various academic perspectives offers similar interpretations of these findings, focusing on the consequences of vulnerable older people's interactions with their less supportive residential contexts. Disability theorists recognize that functional limitations make it more challenging for older people to meet their daily needs. However, they often highlight discordant environmental features (e.g., travel obstacles, insensitive design, uninviting activities) as failing to lessen the impact of these individual impairments. In this way, environments can be just as “disabled” as individuals (42, 43).

Capability theory, developed by A. K. Sen, similarly distinguishes between a person's mental and physical abilities and their “capabilities.” Harmful environmental activities or economic barriers can negatively influence individual capabilities by hindering behaviors or imposing physical or emotional costs. However, older people can experience greater “capabilities” than their functional disabilities suggest if their living environments provide supportive and therapeutic options (44–46).

Powell Lawton's “environmental docility” hypothesis also argues that environmental features have a more pronounced impact on the behaviors of older people with functional or mobility limitations. Restrictive or unsafe physical or social conditions in homes, neighborhoods, or communities are more likely to limit or threaten the actions of vulnerable older adults (47). However, when residential environments are more supportive, they compensate for a decline in individual resources. Consequently, competent behaviors depend on both individual and environmental factors (48).

## Introducing the digital environment

Consistent with these theoretical perspectives and the substantial body of supporting empirical research, rectifying the mismatched residential environments of more vulnerable older adults should lead to more positive and supportive individual-environment interactions. Consequently, when older individuals introduce digital environments into their homes, they will discover new ways to cope with their healthspan gap and health-related stressors, leading to more positive residential experiences, improved health outcomes, and optimal aging.

The digital environment comprises a connected network of internet-enabled devices, sensors, and software applications, often powered by artificial intelligence (AI), that interact virtually with a wide range of digital destinations. The conceptual model separates the digital environment into two interconnected components: its digital equipment and its digital destinations. The former has both a material (e.g., distinguishable equipment features) and a digital presence (e.g., software); the latter, which relies on the former's functionalities, has only a digital presence, but may also have a geographical or physical counterpart. One definition that captures this duality describes the digital environment as “*the whole continuum from the tangible aspects of computing devices, their programming and information systems, the network technologies connecting them, and the product of interactivity between people to people and people to the digital interface*” ((49), p. 2). Each component is described below.

### Home-based digital equipment

The home's digital equipment collects, processes, and communicates information and data to older occupants or their digital destinations. It typically consists of the following (50):

- Broadband/internet connections, routers, sensors, cameras, and other network devices powered by AI algorithms,
- Digital devices or tools such as desktop computers, smartphones, laptops, tablets, and webcams.
- Voice-activated personal digital assistants, such as Amazon Echo or Google Nest, are AI-powered software programs embedded in smartphones, tablets, computers, and smart speakers.
- Executable software applications (apps) and platforms installed by users on their digital devices and accessed with icons, internet browser shortcuts, voice commands (e.g., Siri), and AI assistants (e.g., ChatGPT, Google Gemini). AI increasingly personalizes these applications to align with users' preferences and needs.
- Assistive and social robots, often designed with animal- or human-like features, can understand older people's spoken queries and respond conversationally.

### Home-based digital or virtual destinations

Digital destinations are virtual or online places and spaces that older adults access by activating software applications/platforms on their digital devices through voice commands, taps, motions, or swipe gestures. They serve as the navigational endpoints of online communications, transactions, and activities, where older users interact virtually with people, chatbots, websites, and other digital contexts that provide information, products, services, activities, and social and emotional support. Common “destination” endpoints include e-commerce and banking sites, libraries, healthcare portals, doctors' offices, educational platforms, news media and social media sites, search engines, payment and booking systems, neighborhood forums, self-help groups, entertainment, travel, and other recreational sites. The concept of “digital destination” first appeared in the tourism and hospitality literature, where researchers studied digitally mediated encounters with tourist-focused places and services (51).

Recognizing digital destinations as a distinct category within the digital environment acknowledges that although older people might have multiple apps, platforms, and websites on their devices, their presence does not clearly indicate how important they are to them or how often they use them to reach their virtual destinations to satisfy their unmet residential and health needs. This distinction is also crucial because accessing these destinations digitally introduces its own usability issues (e.g., navigational challenges, visual clarity, different password protection methods, advertisement overload, and concerns about scams).

**Figure 1:**
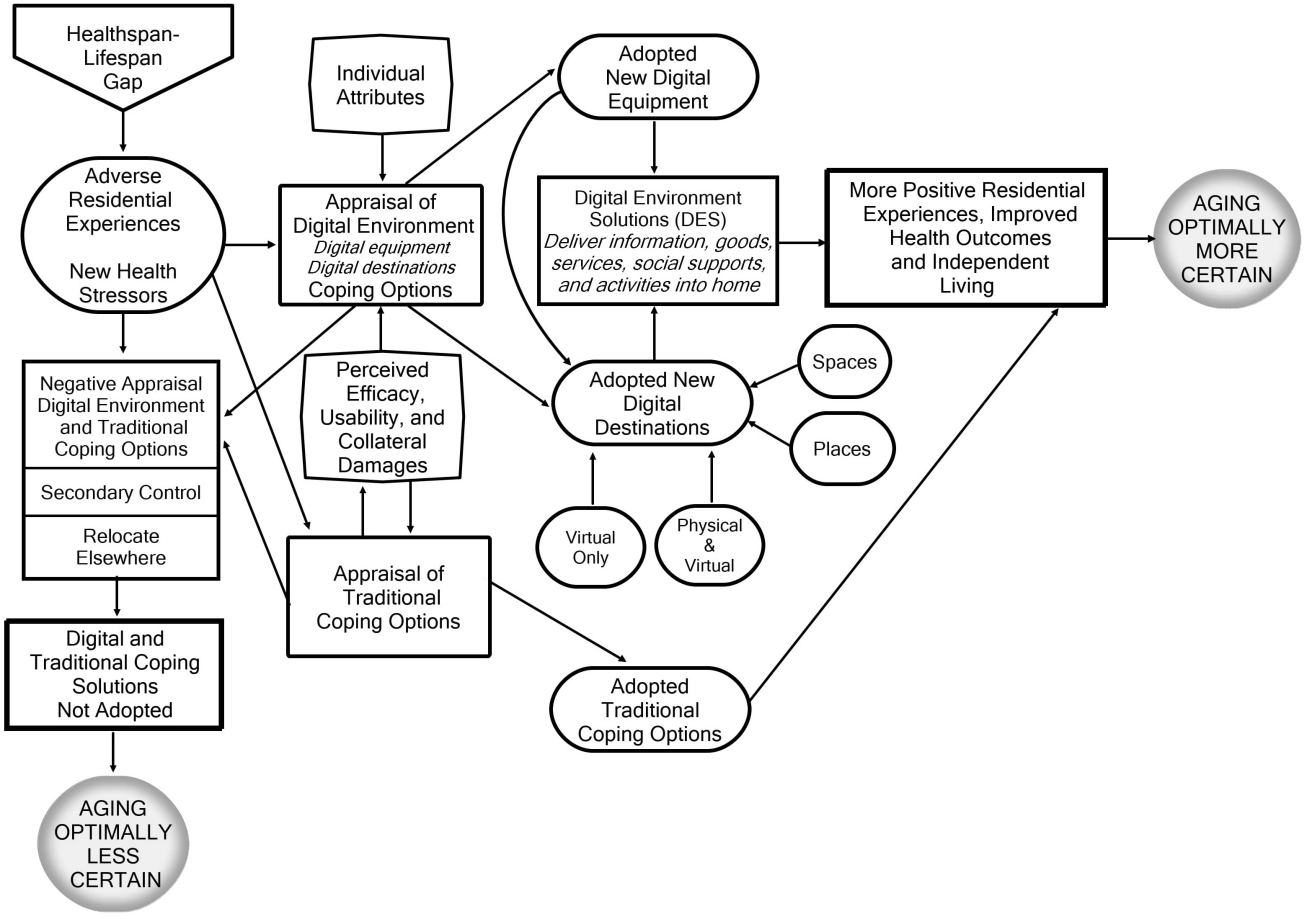
Decision-making pathways influencing whether older people adopt digital environment solutions (DES) and experience positive residential environments and health outcomes, independent living, and age optimally.

Digital destinations can also be categorized by the types of interactions they support (Figure 1). Some appear or function as “spaces,” by offering connections without a human interface or social context. Examples include online retail purchases, depositing funds into a bank account, accessing patient lab results, or obtaining information from news media sites or chatbots. In contrast, other digital destinations serve as “places” that encourage interpersonal social engagement and connections. These include video calls with family via FaceTime, telehealth sessions with doctors or mental health professionals, or participation in online peer or support groups, such as caregiving forums or neighborhood-based organizations. In these “places,” older adults feel a sense of attachment, belonging, and shared identity with their participants (52).

Digital destinations can be entirely virtual or hybrid, the latter combining virtual and physical elements. Fully virtual destinations have no physical counterpart. That is, they do not exist “alongside” physical or geographically located establishments that enable in-person access. Examples include many e-commerce sites, financial platforms, media outlets, and AI-powered information services. For instance, activities such as shopping on Amazon, communicating with a chatbot, streaming news, or attending online mental health sessions typically occur only in a digital environment.

Hybrid digital destinations, by contrast, combine online access with a geographic or physical presence (53). Examples include big-box retailers like Walmart, where merchandise can be purchased both in physical stores and online; FaceTime platforms connecting users with family members in their actual homes; or libraries that lend both on-site physical books and digital e-books. Probably, the most visible and popular hybrid digital destinations are restaurants and fast food outlets, which now almost always have both a physical and a virtual presence. Such digital transaction endpoints blur the line between virtual and physical worlds, enabling older adults to experience continuity across both. Recognizing these hybrid destinations also suggests a new quality-of-life indicator for neighborhoods and communities, highly relevant to older adults. That is, when the stores, offices, restaurants, people, organizations, and activities they can access digitally also have physical locations in their neighborhoods and larger communities, older people will arguably evaluate them more favorably than comparable entities with only a digital presence. For example, they would be more likely to patronize a physician or psychologist who offers telehealth services and has a community-based office, and they would prefer neighborhood-based support groups with both an online and a physical presence (54).

## Digital environment solutions

Introducing these digital environment components into their homes makes it possible for older individuals to adopt as many as the following four categories of digital environment solutions (DES):

### Healthier, safer, less lonely, and more supportive homes

When equipped with internet connectivity and digital devices, the home becomes a hub for environmental and individual monitoring. Sensors embedded in walls, floors, appliances, and furnishings record, store, and analyze data about a dwelling's interior environment, such as air quality and temperature, thereby reducing health risks. iPhones and wearable devices, such as smartwatches, rings, and glasses—often powered by AI—continuously monitor individuals' medication use, heart rate, blood pressure, glucose levels, fitness, movement patterns, and sleep behaviors (aka, digital phenotyping) (55). By generating real-time feedback and reminders, they support adherence to medical treatment, enable early detection of emerging physical health conditions, reduce the need for caregiver visits, reduce medication errors and emergency room visits, and help prevent hospitalizations. In addition to communicating findings to the home's occupants, many systems transmit data to secure cloud platforms, where healthcare professionals and mental health counselors can provide timely guidance (51, 56, 57).

Advanced motion and sensor detector systems in a dwelling's doors, walls, and ceilings can also monitor individual movements or behaviors that signal health concerns, such as prolonged periods of inactivity, nocturnal wandering, or reduced time spent in specific areas, such as kitchens or bathrooms (51). Similarly, fall-detection devices alert family members or emergency services, providing essential rapid responses (e.g., for a stroke victim). The most unobtrusive monitoring technologies operate passively, requiring no active input from older users—an essential feature when individuals are unable to communicate their needs in medical emergencies. Older people feel emotionally more secure, safer, and confident in their homes, knowing that health alerts will be communicated promptly. Aging in place and living independently become more feasible.

Socially assistive robots, such as Paro the seal, Bono, JustoCat, and Huggable, offer a very different category of solutions—companionship and emotional support. These pet-like robots respond to user verbal cues, learn from prior interactions, and provide increasingly personalized (AI) interactions (58).

Emerging home-based humanoid robots (e.g., Neo) offer much more than companionship—performing household chores, preparing meals, providing information, monitoring medications, and providing assistive support and cognitive stimulation (58, 59).

### Access to health care digital destinations

Telehealth (also known as telemedicine) is among the most impactful digital solutions for improving the health outcomes of older adults. Online consultations—delivered through platforms such as Zoom—allow physicians, nurses, physician assistants, mental health counselors, and nutritionists to provide care remotely. Family members can participate in these sessions, thereby enhancing communication and shared decision-making (60). These virtual visits serve many of the same functions as in-person care, including diagnosis, monitoring, counseling, and treatment. Patient portals further expand access by enabling older adults to review their laboratory results and medical records from the comfort of their own homes. Additionally, physicians across different medical centers can share this information to coordinate the care of older patients.

While not suitable for procedures requiring hands-on intervention, telemedicine is widely recognized for its potential to manage chronic conditions and multimorbidity, particularly when used in conjunction with in-person care. Evidence shows that telehealth visits reduce missed appointments and decrease exposure to contagious illnesses—benefits especially critical for immunocompromised individuals (61). Other virtual sessions with mental health counselors extend access to therapy, reducing barriers for those managing depression and anxiety.

Digital destinations also provide abundant health information, enabling older adults to compare treatment options, learn about prevention, and enhance their eHealth literacy. AI-powered personalization further tailors these resources to each user's unique health profile. Also significant are online support groups and forums where older adults share experiences, discuss coping strategies for chronic illnesses, and connect with peers dealing with similar challenges, including caregiving stresses and the symptoms of Alzheimer's disease (37). Because of these digital resources, older adults' awareness of their health needs increases, and they take more responsibility for their self-care.

### Access to digital destinations for information, goods, services, and activities

The proliferation of digital destinations, often tailored to individual users, also expands access to a wide range of goods, services, and information that previously required physical travel. Common examples include AI-powered chatbots that can answer questions and E-Commerce platforms that supply clothing, groceries, medications, and household items, often with detailed product information. Online banking and investment portals provide convenient transaction services, fostering confidence in managing financial independence. Access to these digital destinations increases awareness of helpful resources, promotes healthier lifestyles, increases feelings of self-efficacy, and helps older adults feel more confident in living independently (51). Educational platforms, online courses, and brain-training sites provide information that further supports mental engagement (preventing cognitive decline) and promotes lifelong learning, thereby boosting feelings of competence (62).

Also important are digital destinations with engaging content that satisfy discretionary needs (63). Streaming recreational services, online libraries, and cultural experiences—including virtual museum tours—transform homes into entertainment hubs, fostering engagement, stimulation, and enjoyment (40, 64). Emerging immersive technologies, such as augmented reality, further expand opportunities for exploration and leisure (51).

### Access to digital destinations for social support

A fourth category of solutions enables increased social engagement with family, friends, neighbors, and new acquaintances—particularly younger generations. Early evidence from the COVID-19 pandemic suggested that digital transactions can reduce social isolation and loneliness, sustain existing social networks, and foster a sense of belonging (52, 65). Findings also show that these digital solutions help older adults participate in online social activities with others who share similar interests (16). These social connections provide beneficial instrumental and emotional supports, reduced anxiety, a greater sense of purpose, increased self-efficacy, and cognitive stimulation (4, 66, 67). Key digital destinations include:

- Digital messaging and video calls (e.g., Zoom, Facebook), especially with people who offer opportunities for rich storytelling and photo sharing.
- Social networking sites, virtual reality spaces, and online interest groups offer opportunities for older adults to volunteer, mentor, or engage in peer communities.
- Senior center websites and targeted portals offering classes, wellness programs, and social events (68). Some of these digital destinations offer programs designed to enhance older adults' digital literacy.

## Consequences and influences of digital environment solutions

### Individual outcomes

The model proposes that when older adults adopt and continue using these solutions (69), they have more positive residential experiences and improved physical, psychological, and social health outcomes. They gain new ways to avoid or mitigate adverse interactions with their external environments, such as travel barriers that limit access to neighborhood and community destinations (49, 70). Their homes become more protective—healthier, safer, and more supportive—environments, and they are better prepared to handle the limitations of home-centered lifestyles. Overall, they feel more confident about aging in place independently—avoiding relocation to places that offer more assistance and care—even as they rely less on family and paid caregivers (71).

Because these technologies are central to an older person's overall quality of life, the model links these adoption behaviors to the broader goal of aging well. However, it equates aging well with “aging optimally”—the best possible way under given circumstances (9)—rather than with the traditional framing of “aging successfully” (72). This distinction is critical. The “successful aging” model emphasizes three benchmarks: avoiding disease and disability, maintaining cognitive and physical functioning, and sustaining social engagement. Yet, measured against these criteria, only a small proportion of older adults would be aging successfully. Moreover, its reliance on individual health and activity indicators neglects the role of residential environments in supporting or hindering well-being (73).

By contrast, interpreting aging well as meaning to age optimally draws attention to the importance of subjective aging—how individuals perceive and evaluate their own experiences of growing older (74). This perspective aligns with lifespan developmental psychology, which emphasizes that older people proactively set goals, adapt to constraints, and pursue opportunities in diverse ways. Focusing on optimal aging highlights that the “good life” (47) is not universally defined but rather deeply personal, which helps explain why older adults cope differently—seeking individually tailored outcomes—when facing challenges to their personal health or housing environments.

### Individual attributes influencing appraisals of digital environment coping options

Adverse personal circumstances resulting from age-related health issues (a consequence of the healthspan-lifespan gap) and their mismatched living situations and health stressors (29, 75) mainly motivate older adults to adopt and sustain the use of these digital solutions (69). However, older adults will not respond equally to these influences, and some will more proactively seek solutions because managing their daily activities and health problems exceeds or strains their abilities or resources, threatening their quality of life (47, 75). These individuals are especially stressed when their health conditions are chronic—such as ongoing back pain or muscle weakness—and progressive—such as Parkinson's disease or dementia—rather than if their effects are temporary, like during recovery from knee surgery (76).

Several other individual factors also predict whether older people are motivated or able to cope with these individual stresses. Personality differences can play a central role. More resilient older people are more inclined to seek solutions to their problems. They possess the “*capacity to overcome, steer through, and bounce back from adversity*” ((77), p. 1782). They believe more strongly in their abilities to accomplish necessary tasks (78). Faced with challenges, they focus on their strengths rather than their weaknesses. Thus, they are more confident in their ability to take control of their lives or their environment—and are more ready to act.

Resilience also fosters that openness to new and unfamiliar experiences, which is essential to considering innovative technologies. Older individuals with more venturesome personalities are more willing to abandon entrenched habits, experiment, and embrace the potential of new technologies to mitigate the effects of their limitations (69, 79, 80). These individuals are less intimidated by unfamiliar, seemingly complex tech products and are more willing to accept the risk of making mistakes, which tech users often experience (81, 82).

Economic factors often play a decisive role. When older adults are less financially constrained, it becomes more feasible for them to purchase, install, and maintain digital devices (69). Access to many digital destinations is also more achievable because they often charge subscription or delivery fees to access their information, goods, or services.

Cultural and religious factors also shape adoption decisions. Language barriers may prevent ethnic minorities, immigrants, or members of indigenous communities from accessing information about their digital options because of limited English proficiency or illiteracy in their own language. Older people also report that specific digital destinations are culturally inappropriate because of their insensitive content or approaches (e.g., triggering past traumas) (27, 63, 83). In some cultural subgroups, a strong belief that families should assume caregiving responsibilities causes older adults to dismiss technological alternatives as unnecessary. For example, they may consider monitoring a vulnerable older person to be the responsibility of the extended family (84). They might also argue that traditional caregiving practices, solutions, and travel access are more effective than digital solutions (85). However, opposite patterns also exist: some older adults actively use information and communication technologies to deepen their spiritual and religious engagement (86).

The presence of physical and functional health impairments—such as dexterity or activity limitations—may leave older adults feeling uncertain about their ability to adopt new skills. For individuals with sensory impairments and reduced cognitive abilities, including diminished attention, reasoning, or problem-solving skills, new technologies—such as digital devices or navigating websites—may seem especially intimidating to engage with and use (62, 78, 87, 88).

Gerontological researchers have long depended on these competence indicators to explain differences in older people's ability to maintain their independent living arrangements and age in place successfully. However, as the digital environment becomes more relevant, there is a greater need to recognize digital literacy as a vital indicator of individual competence, potentially as important as these traditional health and mobility factors (89). When older adults are more digitally literate, they will have more complete and accurate information about the usefulness—that is, the degree to which it is rewarding and beneficial—of their digital equipment and digital destination choices, and about the digital environment solutions they enable. They will be more alert to risks such as privacy and criminal intrusions. Overall, they will be able to access and navigate their digital environments more effectively and with greater confidence.

Explaining individual differences in digital literacy is itself a subject for analysis. Health or disability limitations, lower educational backgrounds, and low income are often causes, but location of residence (e.g., country or urban-rural settings) and lack of broadband access or other equipment deficiencies can also be important factors. Other influences include older people's past experiences with technology (i.e., “internal information”). Because of their work histories, for example, they now may have more skills, confidence, and experience with using digital devices and accessing digital destinations (69).

Older individuals' digital literacy also depends on the quality of information from external sources, including people and organizations (85, 90). This information tends to influence their judgments more when it comes from credible, trustworthy, and unbiased sources that they find relevant, timely, and easy to understand (63, 91, 92). Close friends, respected service providers, professionals, and familiar media spokespersons all fall into this category. However, some experts argue that healthcare professionals should increasingly take on these educational roles (93).

Other older people will feel most comfortable learning from younger generations. They will seek guidance from tech-savvy adult children or grandchildren (78, 94). Less intimidating and judgmental, these key figures can help reduce anxieties about using digital devices and explain the benefits and functions of specific digital destinations.

When these sources of information are unavailable, older adults often benefit from digital literacy classes offered by community-based senior centers and public libraries, especially those with an online presence. Traveling to these places would be a better solution, but then they would face the usual physical accessibility challenges (93).

### Coping appraisals of digital environment coping options

Building on extensive prior literature on technology adoption, the model links older people's adoption behaviors to how they assess the efficacy (usefulness) and usability of digital environment technological options (10, 12, 69), along with their unintended consequences (collateral damages) such as privacy breaches, criminal intrusions, and lost human connections (11).

#### Efficacy of digital environment coping options

Influenced by individual attributes and available information, older people will evaluate whether potential digital devices and digital destinations can effectively address their unmet residential needs and health challenges. They must question whether it is in their best interests to rely on these solutions to live in safer and healthier dwellings and to deliver the goods, services, information, social supports, and activities they need. They must weigh the advantages and disadvantages—or perceived effectiveness—of adopting these solutions. For example, they may prefer not to be monitored by video sensors rather than motion detectors, or opt for smartphones over iPads, or consider both devices less effective than personal digital assistants. Alternatively, they may be more enthusiastic about smart watches to monitor their vital signs. They may judge specific digital destinations as more effective than others for achieving improved physical or emotional health outcomes. Societal contexts can matter. During the COVID-19 pandemic, for example, older adults enthusiastically turned to digital destinations made possible by FaceTime and Zoom to stay connected with loved ones and access telehealth services.

Other appraisals by older people will distinguish between the “places” and “spaces” of digital destinations, favoring those that provide live human interaction rather than impersonal, automated platforms (Figure 1). For instance, some may resist telecounseling digital destinations because a consulting psychologist lacks the physical presence of a local community office. Conversely, older people may view digital destinations ending in spaces as more appealing because they want to avoid others “seeing” their physical vulnerability.

#### Usability of digital environment coping options

However, the perceived effectiveness of their coping options may be insufficient to encourage adoption. Usability issues may be more important. Older adults may live in less urbanized countries or communities that have poor-quality or nonexistent broadband coverage. Those occupying these more rural or remote areas are also disadvantaged when traditional trucking services struggle to deliver products purchased virtually (95). However, older adults may still find these digital solutions more usable if they face greater barriers accessing traditional healthcare coping solutions in these less built-up places.

Other older adults may feel anxious, frustrated, or incompetent when trying to operate certain digital devices or use specific software platforms. Those with sensory impairments may also find the most useful digital destinations too complex or demanding to use, such as complicated-to-navigate physician portals or government service websites. Other adoption barriers occur because some destinations have visually confusing or difficult-to-navigate menus or require multi-step security processes (such as two-factor password authentication). Challenges include entering user information, reading and understanding small-font interfaces, or reaching embedded internet links. They may also dislike that digital destinations bombard them with endless advertising solicitations or having to communicate with humorless, unresponsive chatbots. These issues are exacerbated when older people cannot find user-friendly online help or technical support.

#### Collateral damage of digital environment solutions

Acceptance decisions also depend on older people's fears of suffering collateral damages, that is, the unintended negative consequences of relying on new technological solutions (11). Consequently, they may reject functional and easy-to-use digital destinations if they compromise their privacy or expose them to cyberattacks. For example, older adults worry that their personal data might be misused or that they will be scammed. These threatening external intrusions have not just undesirable financial but also emotional impacts. They also find it troublesome that some digital destinations powered by AI will presume to know their personal preferences.

Older people may feel these digital solutions are dehumanizing and resist being reduced to data streams analyzed by emotionless AI algorithms. They complain that digital spaces lack a human element and are reluctant to give up trusting, reliable, and emotionally close relationships with service providers in local community settings. Even when digital options promise independence and convenience, their impersonal qualities may deter their use.

Perhaps unexpectedly, older people may reject digital solutions because they lead to better health outcomes or greater independence. They work too well. Many oppose devices placed in the home that transform a cozy, familiar environment into a clinical or institutional space (96). Similarly, they worry that AI-enabled monitoring tools, designed to detect troubling behaviors, will interpret unremarkable acts, such as a long afternoon nap, as unsafe or unhealthy. They fear that these problematic behaviors, when shared with family or healthcare professionals, may lead to unwelcome interventions and stigmatize them as disabled or sick and thus burdensome and dependent (97). They particularly worry that such surveillance data might hasten their transition to a family member's home or a long-term care residence.

## The adoption of digital solutions: alternative decision-making pathways

Influenced by their individual attributes and deliberations about their coping options, older people can pursue six alternative decision-making pathways to address the incongruities in their lives and environments (Figure 1).

### Adopters of digital or traditional coping options

Psychologists argue that when confronted with circumstances perceived as stressful, unpleasant, or harmful, most older adults rely on primary control or assimilative strategies to find practical solutions. They try to avoid, eliminate, reduce, or manage the sources of their difficulties (39, 98). In this conceptual model, older adults attempt to improve or better manage their unfavorable residential and health circumstances, thereby increasing their ability to age independently and age optimally (99). Figure 1 identifies three groups of adopters: those selecting digital solutions, those selecting traditional solutions, and those adopting options in both categories.

The first group of older adults will primarily adopt digital environment solutions, but as the previous discussion of their coping appraisal deliberations emphasized, predicting how they made their choices will not be straightforward. For some, digital solutions will be their first choice. Others will have initially evaluated traditional, more familiar solutions favorably but later rejected them and turned to digital solutions. For example, they may have become less confident that they can rely solely on family or neighbors' help. Once focused on these newer solutions, they begin to recognize the efficacy of specific digital tools, such as personal digital assistants or specific digital destinations, but avoid others if they seem too complicated to use or access. Alternatively, they might find some digital destinations helpful and easy to use, but judge the collateral damage risk of others as too great because they fear intrusions on their privacy or have scamming concerns. Financial considerations can play a significant role—a merchant's high delivery costs for an item purchasable on its digital site may be prohibitive, leading them to avoid this digital destination and transact with more affordable ones. These types of tensions highlight the multiple factors that continuously influence their choice of digital environment solutions.

Although the model emphasizes the stress-reduction motivations underlying these choices, older people's coping behaviors may also be motivated by hedonistic reasons (15). That is, they adopt digital solutions in anticipation of the fun, pleasure, or enjoyment (e.g., computer games, virtual recreational sites) they will derive from using a new technology. Yet even these actions may have stress-relieving effects, such as helping older adults cope with boredom or cognitive inactivity.

A second group of older people who experience stressful circumstances will also assess the advantages and disadvantages of their technological options but then dismiss them as unnecessary, unusable, or associated with too many side effects (i.e., collateral damages). However, still determined to find solutions to their problems, they turn to traditional solutions, appraise their efficacy and usability, and compare them with the pros and cons of digital solutions. Based on these deliberations, they ultimately decide to adopt these traditional options (11).

The literature offers many examples of the factors that influence the choices of this second group. For instance, some older adults hesitate to rely on digital technology to replace more valued human connections, and they prefer personal contact with professionals, family members, or neighbors. They feel more confident with the hands-on care they receive during in-person medical visits than during telehealth sessions, and feel more comfortable relying on neighbors for grocery shopping than using online delivery services. Additionally, a lonely, low-income older woman might choose her community's Meals on Wheels program not only because it is more affordable than online alternatives but also because she enjoys the warm social interactions with her delivery driver. Moreover, if family members already help monitor medication adherence or ensure their loved ones' safety at home, older adults often see no need for digital devices that claim to do the same.

The benefits of engaging with their tangible, physical worlds, which they find more familiar and a less intimidating way to access information, goods, services, activities, and social connections, influence others' behaviors. For example, they want to try on clothing in a real department store. They want to touch the fruits and vegetables or see a new kitchen gadget in person. Alternatively, their decision not to adopt digital solutions is shaped by the appeal of new-age-friendly community initiatives, such as improvements in senior centers or the creation of outdoor spaces more appealing to older adults (100).

A third group of older adults, likely the majority, can be accurately called hybrid adopters because they subjectively assess both digital and traditional solutions as providing overall beneficial results. They end up relying on a mix of these old and new options to deal with their stressful situations or hedonistic needs.

### Older persons rejecting both digital and traditional coping options

Three additional categories of older adults (groups 4, 5, and 6) reject both technological and traditional service, care, and transportation options as coping interventions. The fourth group does not feel sufficiently stressed—behaviorally or emotionally—by their individual or environmental adversities to seek remedies. Alternatively, they evaluate the drawbacks of initiating potential coping behaviors and conclude that these outweigh their potential benefits. They are willing to tolerate what they perceive as minimally adverse outcomes in their current lives or environments. Because they do little to rectify their incongruous lives and environments, their experienced residential and health outcomes, and how optimally they age remain uncertain but are likely to skew negative.

A fifth group of older adults copes with their problems mainly through mental responses, known as secondary control or accommodative strategies (98). These individuals downplay or ignore the seriousness of their issues, believing their expectations, goals, or hopes to be unrealistic or overly ambitious. Essentially, they see themselves, rather than their environments, as the problem. Alternatively, they feel overwhelmed by their unfavorable circumstances and believe they have a low chance of finding concrete solutions (101). Others justify their inaction on religious grounds, viewing their situation as part of God's plan, and feel it would be pointless to try to change their lives or environments. The older adults in this group are unlikely to adopt digital or traditional solutions and are at significant risk of experiencing unfavorable personal consequences if they try to age in place in their current homes.

A sixth and final group of older individuals depends on transformative environmental strategies (40). They take proactive steps to address their health issues and housing mismatches by moving from their current homes to more supportive residential or care environments—such as living with family members, opting for shared living arrangements, or choosing planned senior housing and care options. They may benefit from either digital or traditional supportive care solutions in these new settings, but whether these moves result in better outcomes and optimal aging is uncertain and is a question for empirical research.

## Discussion

Older adults now expect more from their homes than ever before (24). Despite age-related declines and losses, most resist moving into the residences of their families or transitioning to senior care communities. They prefer to age in place. They aim to maintain their independent lifestyles, wanting their residences to serve as supportive environments that enable them to manage chronic health and mobility issues, new health stressors, social disruptions, and challenges to accessing their outside worlds (21).

Advances in digital technologies powered by artificial intelligence have made these goals more attainable, allowing older adults to access up to four categories of digital environment solutions. Sensors in homes and wearables can reliably monitor their health and lifestyles and respond to serious falls, medical emergencies, or unusual behaviors. Social robots increasingly provide emotional support. Homes virtually connected to digital destinations help older adults mitigate or compensate for the negative impacts of their home-centered lifestyles and reduce dependence on traveling to often inaccessible or inhospitable outside locales. As a result, older adults now have new ways to access a broad range of resources without leaving their dwellings, including information, retail goods, food and groceries, entertainment, education, healthcare, financial and government services, technical support, and social interactions with family and friends.

Integrating these digital solutions into their homes signifies a paradigm shift in how more vulnerable older adults can cope with their adversities and adapt to their current circumstances. Their homes are no longer merely unconnected, static physical sites but have evolved into dynamic environmental monitoring hubs and digital control centers that foster a sense of competence and control over their lives and surroundings—an essential aspect of achieving “residential normalcy” (21, 40). These new coping options are empowering, enabling older persons to make their dwellings safe, secure, and supportive, and to access their essential and everyday needs despite their spatially restricted activity spaces. With these new adaptational strategies, it becomes more likely that aging in place will be equated with “aging in the right place” (102) and aging optimally (9).

Therefore, understanding the factors behind older adults' decisions to adopt digital technologies is essential. The ELDERDES conceptual model developed in this paper aims to improve on previous efforts in several aspects. It conceptualizes a digital environment introduced into older people's homes, dividing it into two distinct parts—digital equipment and digital destinations—and highlights six decision-making pathways that older adults follow when considering the use of these digital solutions. This enables the model to focus on the conflicting motivations and abilities that shape older people's willingness or hesitation to adopt.

The model emphasizes that older adults are not a homogeneous group. They differ in how proactively they cope with the disconnects in their lives and environments, and individual differences help explain their decision-making. Consequently, they assess the effectiveness and usability of their digital options differently, and some are especially deterred by privacy threats and fears of being scammed. The model focuses on traditional personal attributes such as personality, income, health, religious beliefs, and culture, but additionally argues that digital literacy has become a new competency indicator, shaping the motivation and ability of older adults to adopt these digital solutions.

Unlike most prior analyses of technology adoption decisions, this model recognizes that older adults' digital adoption decisions are harder to predict because they are not made in a competitive vacuum. Many evaluate and compare traditional care and service options with digital solutions for their health or long-term care needs, but then adopt the more familiar options. They may find telehealth visits unacceptable substitutes for in-person care and feel uncomfortable with medication reminders from a personal digital assistant. They are less confident that adopting digital solutions will lead to better individual or residential outcomes than relying on the assistance of a trusted family member. More research is needed to determine whether favoring digital solutions over traditional ones, or vice versa, will achieve comparable benefits or outcomes, and there is currently no theoretical basis to support either position.

These advances in digital environments also call for a reassessment of traditional theories about individual-environment relationships. For example, Lawton's “environmental docility hypothesis,” which suggests that physically or mobility-limited older adults are more susceptible to negative environmental features, might become—fortunately—less reliable in predicting declines in well-being. By becoming digitally literate, older adults can often compensate for personal and environmental constraints and proactively achieve their everyday goals and needs (103, 104).

Despite its improvements, the model presented in this paper has three limitations. First, it remains a linear, static view of technology adoption decisions. In reality, older adults constantly process new information, and their decision-making often involves moving back and forth between options, reappraising choices in light of new internet connectivity, emerging devices, new digital destinations, and varying risks and benefits. For instance, their deliberations might change as AI-powered voice-activated devices become more personalized and housed in friendly humanoid forms. The fluctuating cost of using these technologies can also influence their assessments as they become more or less covered by government programs or insurance coverage.

Similarly, traditional support options may become more or less dependable. Rising private caregiver costs might reduce the feasibility of home care programs. Family members, a key caregiving resource, may become less available or less capable. Home care workers might also be less accessible, effective, and affordable.

Older adults' ability to connect with the outside world will also continually change. Travel access to goods and services varies with store and clinic openings and closures. Advances in vehicle safety, lower-cost ridesharing, and driverless cars may make physical destinations more reachable. In this evolving landscape, older adults will frequently reassess their coping strategies and reevaluate the desirability of both digital and traditional options.

Furthermore, personal motivations for adopting technology are constantly shifting. Older adults' health status and stress levels fluctuate, sometimes improving, sometimes worsening. Unlike physical abilities, digital literacy is a modifiable individual attribute. Additionally, future older cohorts will likely have greater digital proficiency and thus perceive the pros and cons of their digital options differently.

Second, framing digital adoption as simple yes-no choices oversimplifies the complex outcomes involved. Some older adults will adopt digital tools but use them infrequently; other adopters may find their devices or destinations difficult to operate, struggling to understand their features. Some may become discouraged by their limitations, while others feel empowered by their new options. Adoption will also expose individuals to the risks of sharing personal data with unscrupulous entities, leading them to use digital solutions less frequently.

Third, there is a risk of overestimating the potential of these digital solutions. They likely will not meet the needs of the most vulnerable, such as those with severe mobility impairments or advanced dementia, who require constant in-person support and regularly travel to healthcare facilities. Assisted living or nursing homes might be more appropriate for these individuals. Additionally, digital solutions do not address other troublesome housing issues, such as unaffordability, major repair needs, or the lack of age-ready design features.

Finding practical, acceptable solutions to minimize adverse outcomes of the healthspan gap will likely remain a significant challenge. Just as age-friendly initiatives, like those promoted by the World Health Organization, focus on making neighborhoods and communities more accessible for daily activities, we will need new digital strategies to help vulnerable older populations live in safe, healthy homes and connect virtually to destinations that meet their material and social needs. There will be increasing urgency to shift priorities, whereby we are not only creating physical environments that empower older adults but also making digital environments more available to older adults that do the same. This will require making the digital equipment, digital destinations, and digital solutions found in the homes of older adults more age-friendly. Such efforts must become a more prominent focus of research and intervention than adapting physical or social environments to enhance age-friendliness.

Understanding and influencing the factors that motivate and enable older adults to adopt these new technologies will require coordinated efforts across disciplines, including community planners, psychologists, gerontologists, engineers, designers, healthcare workers, and social workers (105). Widespread adoption will also depend on equitable, inclusive policies that address digital literacy, protect privacy and security, and ensure affordability.
